# Adipogenin-seipin, lipid droplet architecture and the expanding metabolic frontier: Implications for metabolic disorders and cancer

**DOI:** 10.1016/j.metop.2025.100439

**Published:** 2025-12-23

**Authors:** Maria Dalamaga

**Affiliations:** Department of Biological Chemistry, School of Medicine, National and Kapodistrian University of Athens, 75 Mikras Asias, 11527, Athens, Greece

**Keywords:** Adipogenin, Cancer, Lipid droplet, Obesity, Seipin

## Abstract

Recent work by Li et al. identifying adipogenin as a structural cofactor of seipin introduces a new paradigm in lipid droplet (LD) biology, shifting attention from enzymatic control of lipid synthesis toward organelle architecture as a determinant of metabolic disease. By stabilizing a dodecameric seipin complex, adipogenin redirects triacylglycerol flux from LD nucleation to droplet expansion, thereby promoting LD enlargement, adipocyte hypertrophy and adipose tissue growth. This mechanism refines the adipose tissue expandability hypothesis by highlighting the importance of endoplasmic reticulum–LD interfaces and their associated microproteins in determining lipid storage capacity. Beyond adipose tissue, LDs have emerged as multifunctional organelles in cancer, supporting metabolic flexibility, redox homeostasis, hypoxia adaptation and resistance to cytotoxic therapies. Although adipogenin expression appears restricted to adipocytes, the structural principle it exemplifies, i.e. that small ER-embedded cofactors may modulate seipin assemblies and LD dynamics, may extend to malignant cells through yet-unidentified microproteins. Collectively, these observations position LD architecture as a conceptual model linking obesity, associated metabolic disorders, as well as cancer pathogenesis, suggesting that targeting organelle-level regulation, rather than lipid metabolism alone, may open new avenues for potential therapeutic interventions.

The recent study by Li and colleagues introduces adipogenin as a structural modulator of lipid droplet (LD) formation, reshaping our understanding of adipocyte biology [1]. Adipogenin is a 80-amino acid highly conserved microprotein, found in adipose tissue, steatotic liver and testis. It is induced mainly by the transcription factor PPAR-γ and is associated with serum leptin levels [2,3]. By demonstrating that adipogenin stabilizes a previously unrecognized dodecameric seipin complex, their work moves the field beyond traditional enzymatic and transcriptional frameworks, and toward a subtler but profound concept: organelle architecture as a determinant of metabolic disease [1,4].

This contribution arrives at an important moment. Obesity continues to challenge global health systems, while metabolic reprogramming has emerged as a defining hallmark of cancer [5]. Understanding how cells control lipid storage is central to both fields, but a major missing piece has been the structural machinery that governs the size, number and metabolic capacity of LDs. Li et al. now provide one of the clearest molecular answers to date [1].

## 1 Adipogenin as a structural determinant of lipid droplet growth

Obesity is fundamentally a disorder of lipid storage, marked by the accumulation of triacylglycerols within adipocyte LDs and the expansion of adipose tissue. During adipogenesis, driven largely by transcription factors such as Peroxisome proliferator-activated receptor (PPAR)γ, white adipocytes progress from containing numerous small LDs to forming a single large unilocular droplet, whereas brown adipocytes retain multiple smaller droplets. These morphological transitions are critical because the number and size of LDs dictate both storage capacity and lipolytic dynamics [6,7]. However, despite their physiological importance, the molecular mechanisms that govern LD biogenesis and expansion during adipocyte development have remained incompletely understood.

LDs were once viewed as inert fat stores. They are now recognized as dynamic organelles integrating energy availability, cellular stress responses and membrane biosynthesis [4,8,9]. Seipin, an evolutionary conserved transmembrane protein localized at the endoplasmic-lipid droplet (ER–LD) contact sites, has long been implicated in the biogenesis of LDs, especially in triacylglycerols (TAGs) nucleation, LD expansion and protein targeting [4,10]. Although its regulatory partners remained largely unknown, recent experimental studies have identified several accessory proteins and molecular tethers that interact with seipin at the ER–LD contact sites, including Lipid droplet assembly factor 1 (LDAF1), promethin, and others, which are now recognized as critical for LD assembly and maturation [11].

**Fig. 1:**
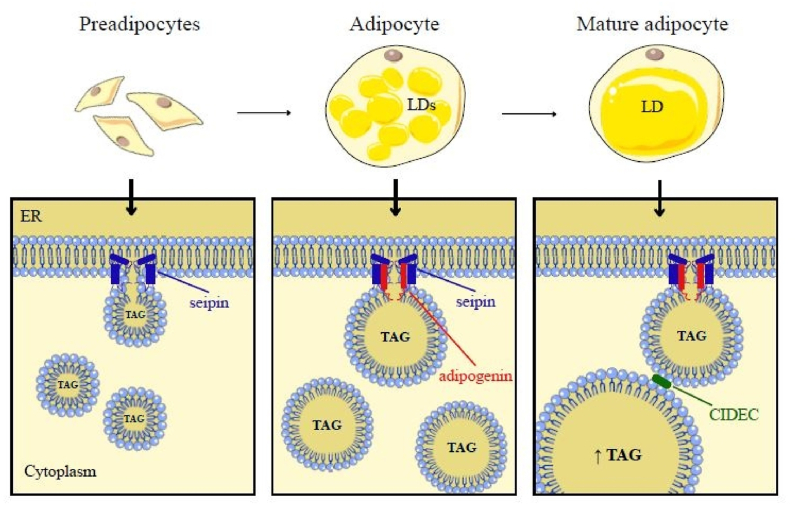
**Legend** Schematic overview of lipid droplet (LD) maturation during adipocyte development and the proposed role of the seipin–adipogenin complex. Preadipocytes contain few LDs. During early adipogenesis, seipin assembles at the endoplasmic reticulum (ER) and assists the formation of small LDs. As adipocytes mature, adipogenin becomes incorporated into seipin complexes, forming a dodecameric structure. This transition is associated with enlargement of existing LDs, increased triacylglycerol (TAG) accumulation and recruitment of factors such as CIDEC that promote the emergence of a large unilocular droplet characteristic of mature white adipocytes. Parts of the figure are from the free medical site http://smart.servier.com/by Servier licensed under a Creative Commons BY 4.0 License https://creativecommons.org/licenses/by/4.0/(accessed on December 12, 2025).

Using cryoelectron microscopy, Li et al. identified adipogenin as a direct binding partner of seipin and demonstrated that this interaction stabilizes seipin oligomerization, shifting the complex from an undecamer (11 subunits, seipin alone) to a dodecamer (12 subunits, seipin–adipogenin) [1]. Although this structural modification appears subtle, it leads to important functional consequences. Adipogenin-driven conversion to dodecamers suppresses the ability of seipin to nucleate new LDs, thereby prioritizing the enlargement of existing droplets over their generation [12]. At the same time, adipogenin selectively binds the dodecameric form, enhancing its rigidity and reinforcing this functional shift. Importantly, adipogenin expression is induced only at later stages of adipogenesis, functioning as a molecular switch that shifts LD biology from nucleation to growth during adipocyte maturation [12]. Fig. 1 depicts LD formation and maturation during adipocyte development and the proposed role of the seipin–adipogenin complex.

The stabilized dodecameric complex promotes the formation of fewer but larger LDs, reflecting an enhanced flux of triacylglycerol (TAG) into individual droplets [1,13]. This structural change also enhances the ER-to-LD relocation of key lipid-metabolizing enzymes, including Acyl-CoA Synthetase Long-Chain Family Member 3 (ACSL3) and Glycerol-3-Phosphate Acyltransferase 4 (GPAT4), as shown in live-cell trafficking experiments, thereby accelerating local TAG synthesis and LD expansion [14,15]. During early adipogenesis, seipin restrains GPAT3 and GPAT4 to prevent the accumulation of phosphatidic acid, a conical lipid that can hinder LD budding and antagonize PPARγ-driven differentiation [1]. Adipogenin relieves this suppression, allowing GPAT3/4 to localize to nascent droplets and promote TAG synthesis once adipocyte identity is established [1]. The coordinated enrichment of these enzymes at the LD surface accelerates both TAG synthesis and turnover, thereby enhancing LD growth and metabolic activity.

As a result, adipocytes accumulate more TAGs and undergo expansion more readily. In mice, the adipocyte-specific overexpression of adipogenin increases fat mass, a process which depends on the presence of seipin. On the contrary, the deletion of adipogenin decreases the amount of seipin complexes in adipocytes and impairs lipid storage in brown adipose tissue [1]. Interestingly, the enlarged LDs observed in adipogenin-overexpressing adipocytes closely phenocopy, i.e. mimic the appearance of, those seen in adipose-specific seipin deficiency, underscoring the functional antagonism between the two proteins [12]. Moreover, adipogenin-driven LD enlargement is likely to potentiate the activity of cell death-inducing DFF45-like effector C (CIDEC, also known as FSP27), a LD–tethering protein that promotes Ostwald ripening, the biophysical process through which lipids flow from smaller droplets into larger ones. This coordinated transfer of TAGs ultimately drives the formation of the large unilocular LD characteristic of mature white adipocytes [1,12]. Collectively, these findings position adipogenin as a microprotein architect that links ER membrane geometry to LD biogenesis and ultimately shapes metabolic fate.

## 2 Hypertrophy, hyperplasia, and the expandability of adipose tissue

Obesity develops through two distinct processes: adipocyte hypertrophy and hyperplasia. Hypertrophy is associated with inflammation, hypoxia, fibrosis and systemic metabolic dysfunction while hyperplasia is often metabolically protective. Human and animal studies have shown that individuals with a predominance of small, numerous adipocytes (hyperplasia) exhibit better insulin sensitivity and lower rates of metabolic syndrome, whereas those with fewer, larger adipocytes (hypertrophy) are more prone to metabolic dysfunction [16,17]. The ability of subcutaneous adipose tissue to expand via hyperplasia is particularly important for maintaining metabolic health, as it prevents lipid spillover into visceral depots and non-adipose tissues, which is a key driver of lipotoxicity and insulin resistance [18]. However, recent data suggest that the relationship may be context-dependent, as some studies have observed that an increase in small adipocytes during rapid weight gain can be associated with increased visceral and ectopic fat accumulation, indicating that hyperplasia alone does not guarantee protection in all scenarios [19]. Nonetheless, the prevailing consensus is that hyperplastic adipose expansion is generally more metabolically favorable than hypertrophic expansion.

The ability of adipose tissue to expand in a healthy manner depends on a well-coordinated interplay of LD formation, ER stress management, vascularization and extracellular matrix remodeling. The adipogenin–seipin axis participates directly in this process. If LD cannot expand, adipocytes fail to buffer fatty acids effectively. Then, lipids spill over into the liver, skeletal muscle, pancreas and cardiac tissue, causing lipotoxicity and metabolic disease. This is consistent with clinical and experimental evidence from conditions such as seipin-related lipodystrophy, where defective formation of LDs may lead to severe hepatic steatosis, insulin resistance and cardiometabolic complications [20,21]. Beyond lipodystrophy, disruption of seipin-dependent LD architecture has also been implicated in neurodegenerative and neuromuscular disorders. Gain-of-function mutations in *BSCL2* cause seipinopathies, including distal hereditary motor neuropathy and Silver syndrome, in which misfolded seipin induces endoplasmic reticulum stress rather than defective lipid storage [22]. In parallel, emerging evidence indicates that LD accumulation and altered LD–organelle contacts in neurons and glial cells modulate oxidative stress responses and neuronal survival. These observations suggest that dysregulated LD homeostasis may represent a shared pathogenic axis linking metabolic, neuromuscular and neurodegenerative disease. Table 1 summarizes human diseases and potential cellular phenotypes associated with seipin (BSCL2) dysfunction.

**Table 1:** Human diseases and cellular phenotypes associated with seipin (BSCL2) dysfunction.

Condition/Phenotype	Underlying Mechanism	Key Clinical or Cellular Features	Supportive Evidence
**Congenital Generalized Lipodystrophy Type 2 (**CGL2, Berardinelli-Seip syndrome**)**	Loss-of-function *BSCL2* mutations → impaired ER–LD contact formation	Near-complete absence of adipose tissue; ectopic lipid deposition; severe insulin resistance; hepatic steatosis; dyslipidemia; cardiomyopathy; sometimes intellectual disability	[20]
**Seipinopathy (motor neuron disease, including distal hereditary motor neuropathy V & Silver syndrome)**	Gain-of-function *BSCL2* mutations → misfolded seipin causing ER stress	Progressive distal muscle weakness; progressive spasticity; motor neuron degeneration; peripheral neuropathy; ER stress-mediated neurodegeneration	[22]
**Celia's Encephalopathy (**Progressive Encephalopathy with/without Lipodystrophy**)**	Cryptic splicing variant (c.985C > T) in BSCL2/seipin	Severe neurodegeneration; progressive encephalopathy, which may be associated with lipodystrophy	[23]
**Metabolic dysfunction (hepatic steatosis, cardiometabolic pathology)**	Inability of LDs to expand → lipid overflow into liver and peripheral tissues	Fatty liver, cardiomyopathy, dyslipidemia	[24]
**Defects in Adipogenesis**	Seipin required for normal adipocyte differentiation and LD maturation	Impaired adipocyte development; failure to form mature unilocular LDs	[25]
**Atherogenesis/Cardiomyopathy**	Seipin deficiency (secondary to lipodystrophy)	Accelerated atherosclerosis, cardiac hypertrophy	[25]
**Cancer-related phenotypes (cellular level)** **A431 carcinoma cells**	Seipin maintains ER–LD contacts, regulating LD number, size, and lipid flux	Increased LD mobility, LD–ER detachment, altered lipid trafficking in carcinoma cell models	[26,27]
**Impaired Brain Development/Spermatogenesis**	Seipin deficiency, aberrant LD homeostasis	Reduced brain volume; impaired memory; defective spermatogenesis and oocyte maturation	[28]

Therefore, the study by Li et al. reinforces the adipose tissue expandability hypothesis, but with a new twist: the limiting factor is not only the capacity for adipogenesis or TAG synthesis, but also the structural stability of the LD–ER interface and the availability of preassembled seipin complexes that enable rapid LD formation [1,29].

## 3 Do similar mechanisms influence cancer cell lipid droplets?

The accumulation of LDs represents a metabolic adaptation process across many tumor types, providing cancer cells with essential energetic, biosynthetic and cytoprotective advantages [30]. Moreover, cancer cells enhance global lipogenic pathways by upregulating enzymes such as fatty acid synthase, stearoyl-CoA desaturase 1, diacylglycerol acyltransferases and perilipin proteins [[31], [32], [33]]. They present markedly increased LD number, size and content compared with normal cells. Enhanced neutral lipid synthesis and lipoprotein uptake rapidly channel TAGs and sterol esters into LDs, supporting proliferation under metabolic stress conditions.

**Fig. 2:**
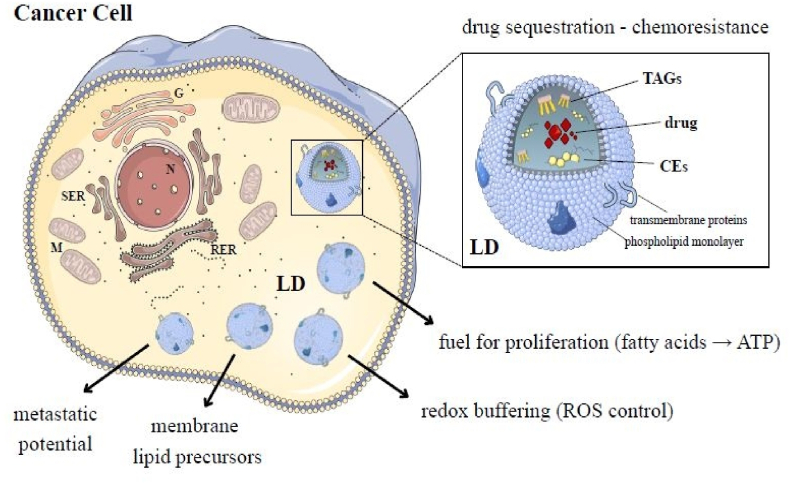
**Legend** LDs as multifunctional organelles supporting cancer cell survival and treatment resistance. The schematic overview illustrates a cancer cell enriched in LDs. These organelles may contribute to tumor fitness by supplying metabolic fuel, providing membrane lipid precursors, buffering oxidative stress and supporting adaptation to hypoxic conditions. LDs may also sequester lipophilic chemotherapeutic agents, reducing their availability at intracellular targets and thereby promoting chemoresistance. The overall accumulation and functional diversification of LDs highlight their emerging importance in cancer pathogenesis and therapy response. Parts of the figure are from the free medical site http://smart.servier.com/by Servier licensed under a Creative Commons BY 4.0 License https://creativecommons.org/licenses/by/4.0/(accessed on December 12, 2025).

Cancer cells exploit LDs to meet several critical demands, including the provision of metabolic energy and biosynthetic precursors, the supply of phospholipids for rapid membrane expansion, the maintenance of redox balance and the sequestration of potentially toxic lipid species [30]. In addition, LDs support cellular adaptation to hypoxic stress and contribute to resistance against cytotoxic therapies [34,35]. Their hydrophobic core can also trap lipophilic anticancer drugs, lowering their effective intracellular concentration and limiting access to nuclear or cytosolic targets, thereby contributing to chemoresistance. Across diverse tumor types, from colorectal cancer and glioblastoma to ovarian carcinoma, elevated LD content may be associated with greater tumor aggressiveness, enhanced metastatic potential and decreased therapeutic responsiveness [36,37]. Fig. 2 depicts LDs as multifunctional organelles supporting cancer cell survival, metastatic potential and treatment resistance.

A key insight from the study by Li et al. is that LD growth may be structurally regulated. This raises an intriguing possibility. Although adipogenin expression appears largely restricted to adipose tissues, it embodies an analogous structural principle. Small ER-embedded cofactors may rigidify seipin complexes and redirect TAG flux, a principle that could extend to cancer cells via unidentified microproteins. Thus, cancer and adipocytes converge on LD expansion, but through distinct enzymatic and structural routes.

Moreover, seipin is broadly expressed across cell types and is known to regulate ER–LD contact sites in cancer cells, underscoring the conservation of this structural machinery beyond adipose tissue [26]. In human cancer cell models, seipin localizes to ER–LD junctions and is essential for the formation, stability, and cargo delivery at these sites, directly influencing LD biogenesis and growth [26,27]. In cancer cells, LDs frequently transition from initial to expanding LDs through localized TAG synthesis at ER–LD bridges, a process supported by Arf1/COPI-dependent trafficking of enzymes such as DGAT2 and GPAT4 [38]. Within this context, the presence of a functionally analogous ER microprotein could plausibly promote LD enlargement, reinforce survival under oxidative stress, accelerate membrane biogenesis and contribute to chemoresistance, mirroring the structural effects observed in adipocytes.

Even if adipogenin itself may be absent in tumors, its mechanism supports the concept that small structural cofactors can fine-tune LD architecture and metabolic resilience. Furthermore, this organelle-level regulation is likely relevant to cancer biology, even though the specific identity of such cofactors in cancer cells remains to be determined.

## 4 Lipid droplet architecture as a therapeutic target

This emerging paradigm may open new avenues in organelle-level regulation and point to several translational opportunities across metabolic and oncologic disease. In obesity, the modulation of adipogenin activity could, in principle, enhance healthy adipose expandability and reduce ectopic lipid deposition, thereby mitigating metabolic dysfunction. Given its restricted expression pattern and association with increased adipose lipid storage, adipogenin may represent a promising therapeutic target for cardiometabolic disorders with minimal systemic effects. However, this approach would require careful calibration, as excessive lipid droplet hypertrophy can impose mechanical and inflammatory stress on adipocytes.

Further work is needed to evaluate whether modulating adipogenin to seipin may alleviate seipin-related diseases (Table 1). In lipodystrophy, where defective LD formation underlies severe metabolic dysfunction, pharmacologic mimetics of adipogenin could theoretically restore LD stability and improve lipid storage capacity, thereby preventing life-threatening complications. In cancer, an almost inverse strategy may hold therapeutic value. Here, inhibiting LD expansion could increase lipotoxicity, amplify oxidative stress and increase tumor susceptibility to cytotoxic therapies [39]. Potential intervention points include disrupting seipin oligomerization, interfering with microprotein–seipin interactions, destabilizing ER–LD contact sites or modulating TAG flux. Supporting this approach, inhibition of LD biogenesis through enzymes such as LPCAT2 or DGAT has been shown to reduce LD accumulation and sensitize cancer cells to chemotherapeutic agents by limiting sequestration of lipophilic drugs [39,40]. Targeting these structural and metabolic nodes may selectively compromise tumor LDs while sparing normal adipose tissue.

## 5 Conclusion

The parallels between adipocyte and cancer cell lipid biology have been noted for years, but adipogenin offers a new conceptual bridge and a significant advance in metabolic cell biology. It shows that the ability of cells to handle lipids is not merely a question of enzymatic supply or transcriptional activation but also of structural organization at the organelle interface. For obesity research, this work reveals a new layer of control over adipocyte hypertrophy and expandability. In adipocytes, adipogenin promotes LD enlargement to store energy safely. For cancer biology, it raises provocative questions about how oncogenic cells might exploit similar mechanisms to support growth and chemoresistance. In cancer cells, increased LD number, size and content allows malignant cells to survive metabolic and oxidative stress. These phenomena reflect two sides of the same structural logic: seipin–microprotein complexes determine how TAG is partitioned, either into a limited number of enlarged LDs, as seen with adipogenin in adipocytes, or into metabolically adaptive LDs in cancer cells. The challenge now is to identify and map microproteins with adipogenin-like functions across tissues, healthy and malignant, and to determine whether manipulating these structural cofactors can reshape disease trajectories. Recognizing this convergence may open new inter-disciplinary strategies, where insights from obesity research inform oncology and vice versa.

## Funding

This work did not receive any specific grant from funding agencies in the public, commercial or not-for-profit sectors.

## Conflicts of interest

No conflict of interest to declare.
